# Review of the genus *Thubana* Walker (Lepidoptera, Lecithoceridae) from China, with description of one new species

**DOI:** 10.3897/zookeys.53.412

**Published:** 2010-08-27

**Authors:** Linlin Yang, Yanmei Zhu, Houhun Li

**Affiliations:** 1 College of Life Sciences, Nankai University, Tianjin 300071, P. R. China; 2 College of Biological Science and Technology, Xinjiang Agricultural and Technical University, Changji 832200, Xinjiang, P. R. China

**Keywords:** Lepidoptera, Torodorinae, Thubana, new species, synonym, China

## Abstract

The genus Thubana Walker is reviewed for China. Nine species are recognized, of which Thubana felinaurita Li, **sp. n.** is described as new; Thubana dialeukos Park, 2003 and Thubana xanthoteles (Meyrick, 1923) are newly recorded for this country; Thubana stenosis (Park, 2003), **syn. n.** is synonymised with Thubana xanthoteles, and Thubana microcera (Gozmány, 1978), **syn. n.** with Thubana leucosphena Meyrick, 1931. Images of adults and genitalia are provided. A checklist of Thubana species in China is included, along with a key to these species.

## Introduction

The genus Thubana was established by Walker (1864) with Thubana bisignatella Walker, 1864 as the type species. Park and Heppner (2009) listed 46 species in their catalogue of the genus. They included Thubana laxata (Meyrick, 1911) and Thubana nodosa (Meyrick, 1910), which were once transferred to Stelechoris (Park and Wu 2001), and Thubana adelella (Walker, 1864), which was earlier removed from the list of Thubana (Park et al. 2005), but did not list Thubana pedicucullata Park, 2009 and Thubana gyrostigmatis Park, 2009 from Philippines (Park 2009), and Thubana reniforma Wu, 2000 from Malaysia (Wu 2000). Here we tentatively add these three species to the list of Thubana.

Prior to this study, seven species of Thubana were recorded in China (Wu 1997). In this paper we describe one new species Thubana felinaurita Li, **sp. n.** on the basis of Chinese material, and report Thubana dialeukos Park, 2003 and Thubana xanthoteles (Meyrick, 1923) as new for China. We synonymise Thubana stenosis Park, 2003, **syn. n.** with Thubana xanthoteles, and Thubana microcera Gozmány, 1978, **syn. n.** with Thubana leucosphena Meyrick, 1931. To date, the genus Thubana comprizes 48 species worldwide including the new species described herein, and nine of them occur in China.

## Material and methods

Genitalia dissections were carried out following the methods described by Li (2002). All the studied specimens, including the types, are deposited in the Insect Collection, College of Life Sciences, Nankai University, Tianjin, China.

## Taxonomic accounts

### Genus Thubana

Walker, 1864

Thubana Walker, 1864: 814. Type species:
Thubana bisignatella Walker, 1864: 814, by original designation.
Titana Walker, 1864: 813. Type species:
Titana adelella Walker, 1864: 814, by original designation.
Tiva Walker, 1864: 821. Type species:
Tiva binotella Walker, 1864: 822, by original designation.
Inapha Walker, 1864: 999. Type species:
Inapha lampronialis Walker, 1864: 999, by original designation.
Stelechoris Meyrick, 1925: 243. Type species:
Pachnistis exoema Meyrick, 1911: 707, by original designation.

#### Diagnosis.

Thubana is characterized by the combination of the following characters: forewing often having a white costal patch, costa gently curved with sharpened end, termen more or less concave, tornus broadly rounded, M3 usually stalked with CuA1+2, R4 and R5 often coincident (insome species R4 and R5 stalked basally), R3 stalked with R4+5; hindwing with M2 present, almost parallel to stalk of M3+CuA1; abdominal tergites with spinose zones; valva thumb-shaped, juxta plate-shaped, and aedeagus with diverse cornuti in male genitalia; antrum cup-shaped, ductus bursae usually with many internal spinules, signum with dense spinules in female genitalia.

Thubana is similar to the genus Torodora in the shape of the wings, the presence of M2 on the hindwing, the spined tergites of the abdomen and the structure of male and female genitalia. But Torodora usually lacks the costal patch on the forewing, and M3 is separated with CuA1+2, which can separate the two genera from each other.

#### Distribution.

China, Thailand, Malaysia, Indonesia, Philippines, India, Nepal, Sri Lanka.

### Checklist of Thubana species in China

#### 1. Thubana albinulla

Wu, 1994

Thubana albinulla Wu, 1994: 130.

##### Distribution.

China (Sichuan).

#### 2. Thubana albiprata

Wu, 1994

Thubana albiprata Wu, 1994: 130.

##### Distribution.

China (Sichuan).

#### 3. Thubana albisignis

(Meyrick, 1914)

Lecithocera albisignis Meyrick, 1914: 50.
Thubana albisignis Meyrick, 1925: 184.

##### Distribution.

China (Taiwan).

#### 4. Thubana bathrocera

Wu, 1997

Thubana bathrocera Wu, 1997: 86.

##### Distribution.

China (Hunan).

#### 5. Thubana deltaspis

Meyrick, 1935

Thubana deltaspis Meyrick, 1935: 563.

##### Distribution.

China (Fujian, Taiwan).

#### 6. Thubana dialeukos

Park, 2003

Thubana dialeukos Park, 2003: 138.

##### Distribution.

China (Yunnan), Thailand.

#### 7. Thubana leucosphena

Meyrick, 1931

Thubana leucosphena Meyrick, 1931: 69.
Thubana microcera Gozmány, 1978: 236, syn. n.

##### Distribution.

China (Anhui, Fujian, Jiangxi, Henan, Hunan, Hubei, Guizhou, Zhejiang).

#### 8. Thubana xanthoteles

(Meyrick, 1923)

Lecithocera xanthoteles Meyrick, 1923: 38
Lecithocera melitopyga Meyrick, 1923: 41
Thubana xanthoteles Clarke, 1965: 232
Thubana stenosis Park, 2003: 147, syn. n.

##### Distribution.

China (Yunnan), Thailand, India, Sri Lanka.

#### 9. Thubana felinaurita

Li sp. n.

##### Distribution.

China (Guangxi).

### Key to male Thubana in China

**Table:** 

1.	Forewing with R4 and R5 short-stalked	2
–	Forewing with R4 and R5 coincident	3
2.	Juxta with posterolateral lobes narrowly rounded at apex; aedeagus with two sclerotized bars, the distal one needlelike, the median one acinaciform (Fig. 9)	Thubana xanthoteles
–	Juxta with posterolateral lobes acute at apex, aedeagus with two to three dentations near apex (Park 2000: Figs 13, 13a)	Thubana albisignis
3.	Forewing without white patch, with small black cell-dot and fold-dot	Thubana albinulla
–	Forewing with white patch	4
4.	Forewing with white basal patch occupying 1/3 of wing, costal patch absent	Thubana bathrocera
–	Forewing without basal patch, costal patch present	5
5.	Male juxta with a long spine at base (Clarke 1965: Figs 2–2b)	Thubana deltaspis
–	Male juxta without spine at base	6
6.	Aedeagus longer than valva (Fig. 7)	Thubana felinaurita sp. n.
–	Aedeagus shorter than valva	7
7.	Juxta without median projection (Fig. 8)	Thubana dialeukos
–	Juxta with median projection	8
8.	Juxta with a lobulated projection at middle, with two large thornlike processes on caudal margin; posterolateral lobes inconspicuous (Fig. 15)	Thubana leucosphena
–	Juxta with a horned projection at middle, without processes on caudal margin; posterolateral lobes mastoid (Wu 1994: Fig. 17)	Thubana albiprata

### Thubana felinaurita

Li sp. n.

urn:lsid:zoobank.org:act:E86B6559-0A44-44A9-A3CD-62F2D3B8ABCF

Figs 1 7 

#### Type material.

Holotype ♂ – China, Guangxi Province: Dongzhong Forestry Farm, Fangchenggang, (21°35'N; 108°22'E), 640 m, 9.IV.2002, coll. Shulian Hao & Huaijun Xue, genitalia slide No. ZYM06312; Paratype– 1 ♂, same data as holotype except dated 8.IV.2002, genitalia slide No.YLL08061.

#### Diagnosis.

The new species is similar to Thubana leucosphena, but can be distinguished from it by the juxta having a median membranous protuberance, the posterolateral lobes rounded apically, and the aedeagus longer than valva. In Thubana leucosphena, the juxta has a lobulate projection at middle near anterior margin and two large thornlike processes on caudal margin, the posterolateral lobes are inconspicuous, and the aedeagus is obviously shorter than the valva.

**Figures 1-6:**
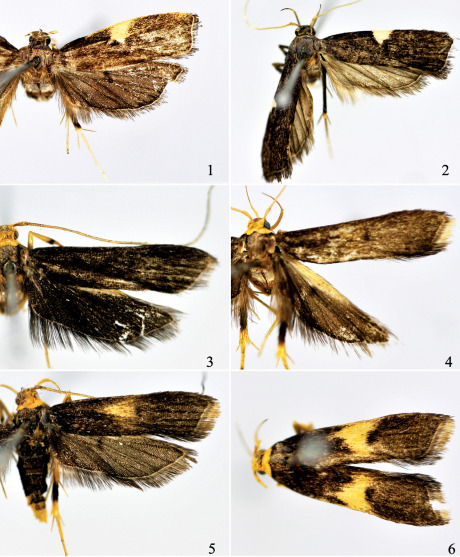
Adults of Thubana spp. **1** Thubana felinaurita sp. n., paratype **2** Thubana dialeukos Park **3–6** Thubana xanthoteles (Meyrick), showing variation of costal patch (1–4 >, 5–6 +).

**Figures 7-10:**
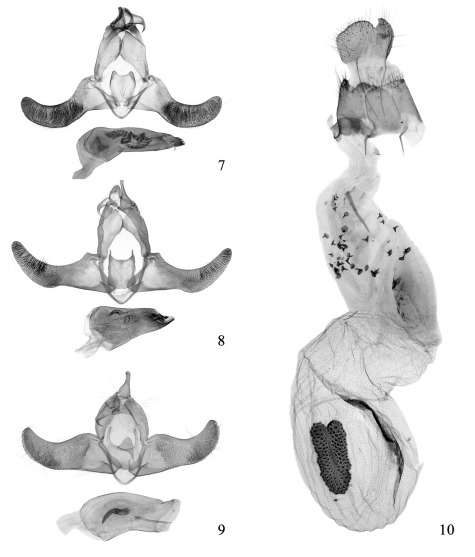
Genitalia of Thubana spp. **7** Thubana felinaurita sp. n., paratype, slide No. YLL08061 > **8** Thubana dialeukos Park, slide No. YLL08075 > **9** Thubana xanthoteles (Meyrick), slide No. ZYM06119 > **10** Thubana xanthoteles (Meyrick), slide No. ZYM06121 +.

#### Description.

Adult (Fig. 1). Wingspan 20.5–21.0 mm. Head grayish brown on vertex, with grayish white scales around eyes. Antenna yellowish white, longer than forewing. Labial palpus brown; inner surface of second segment yellowish white mixed with grayish scales; third segment dark fuscous, longer than second. Thorax and tegula grayish brown, with shining luster. Forewing rectangular, costa gently curved, apex blunt, termen slightly concave inward at about 1/3; color brown with dark purple; costal patch triangular, yellowish white, extending to middle of cell; fringe grayish black, with yellowish white basal line. Hindwing grayish brown; fringe fawn black, with yellowish white basal line. Fore leg with dorsal surface dark grayish, ventral surface yellowish white; mid leg yellowish white, with scattered brown scales; hind leg yellowish white on inner surface, grayish brown on outside except tarsus and distal end of tibia yellowish white.

#### Male genitalia

(Fig. 7). Uncus relatively stout, broad basally, narrowed to bluntly rounded apex. Gnathos large, broad in basal 2/3, strongly bent beyond basal 2/3, then narrowed toward apex; apex hooked, greatly curved ventrally. Valva broad at base, slightly narrowed to basal 1/3; distal 2/3 curved upward like a finger, with dense setae on inner surface; apex rounded; costa protruded basally, incurved medially; ventral margin concave inward at basal 1/3, gently arched outward medially. Sacculus weakly sclerotized, broad at base, narrowed distally, straight ventrally, terminating at basal 1/4 length of valva. Juxta quadrate, with a membranous protuberance at middle; posterolateral lobes like cat’s ear in shape, setose, rounded posteriorly. Vinculum narrow, weakly sclerotized. Aedeagus very stout, longer than valva, broad in basal 2/5, narrowed toward apex, with caducous setae at apex; vesica slightly sclerotized at base, medially with a bundle of numerous brushlike spines, a slightly arched plate and a slender dentate band.

#### Female.

Unknown.

#### Distribution.

China (Guangxi).

#### Etymology.

The specific name is derived from the Latin felinus (= feline) and auritus (= auricular), referring to the shape of the posterolateral lobes of the juxta.

### Thubana dialeukos

Park, 2003

Figs 2 8

Thubana dialeukos Park, 2003: 138.

#### Material examined.

1 ♂, China, Yunnan Province: Rare Botanical Garden, Ruili (24°00'N; 97°50'E), 1000 m, 6.VIII.2005, coll. Yingdang Ren, genitalia slide No. YLL08075.

#### Diagnosis.

This species is very close to Thubana leucosphena and hardly distinguishable from the latter by the superficial characters (Fig. 2) and the venation. However, it can be easily differentiated by the following characters of the male genitalia (Fig. 8): the valva broad at base, narrowed to before middle, gently raising obliquely upward in distal half, narrowed to blunt apex; the costa straight in basal 1/6, gently concave in distal 5/6; the juxta quadrate, with small, slender, almost straight posterolateral lobes; the aedeagus stout, shorter and broader than valva, with two dentate preapical lobes, and the cornuti consisting of a S-shaped fragment and a mass of short spines.

#### Distribution.

China (Yunnan), Thailand.

#### Notes.

This species is recorded for the first time in China.

### Thubana xanthoteles

(Meyrick, 1923)

Figs 3–6 9, 10

Lecithocera xanthoteles  Meyrick, 1923: 38.
Lecithocera melitopyga  Meyrick, 1923: 41; Clarke, 1965: 232, as synonym of Thubana xanthoteles.
Thubana xanthoteles  (Meyrick, 1923): Clarke, 1965: 232.
Thubana stenosis  Park, 2003: 147, syn. n.

#### Material examined.

1 ♂, 7 ♀, China, Yunnan Province: Mengla (21°29'N; 101°33'E), 650 m, 23–25.VIII.2005, coll. Yingdang Ren; 2 ♂, 1 ♀, Jinghong (22°01'N; 100°48'E), 585 m, 17–18.IV.1995, coll. Hongjian Wang & Guangyun Yan.

#### Diagnosis.

This species can easily be separated from its allies by the elongate narrow forewing without patch (Figs 3, 4), or with an orange-yellow cuneate (Fig. 5) or bandlike costal patch (Fig. 6); in male genitalia (Fig. 9), the juxta with digitate, setose posterolateral lobes and a large triangular median projection, the aedeagus as long as and broader than valva, and the cornuti consisting of two sclerotized bars: the distal one needlelike, about 1/2 length of aedeagus, the median one stouter, somewhat acinaciform; in female genitalia (Fig. 10), the caudal margin of 8th sternite deeply emarginate at middle, the ostium broad, the antrum fan-shaped and weakly sclerotized, the ductus bursae narrowed basally, with many short spines medially, and the signum strawberry-shaped.

#### Distribution.

China (Yunnan), Thailand, India, Sri Lanka.

#### Discussion.

Meyrick (1923) described Thubana xanthoteles on the basis of two female specimens and described Thubana melitopyga from one female specimen. Clarke (1965) regarded Thubana melitopyga as a junior synonymof Thubana xanthoteles. Thus previously only three female specimens of Thubana xanthoteles have been known and none of these has the costal patch on the forewing. Park (2003) described Thubana stenosis on the basis of the specimens collected from north Thailand, which bears the “golden yellow bandlike costal patch” on the forewing. He also noticed that the “female genitalia” of Thubana stenosis “are hardly distinguishable from those of Thubana xanthoteles”. In this study, we found that the male genitalia of the three specimens collected in south Yunnan undoubtedly match with those of Thubana stenosis described by Park, and the female genitalia match with those of Thubana stenosis and of Thubana xanthoteles. We also found deciduous needlelike cornuti in the ductus bursae of female genitalia. Superficially, the males have no costal patch, but the females usually have a bandlike or cuneate costal patch. Thus we treat Thubana stenosis as a junior synonymof Thubana xanthoteles, and regard the presence or absence of the costal patch as intraspecific variation.

#### Notes.

This species is recorded for the first time in China.

### Thubana leucosphena

Meyrick, 1931

Figs 11 16

Thubana leucosphena Meyrick, 1931: 69; Clarke, 1965: 231; Gozmány, 1978: 235; Wu, 1997: 84; Park, 2003: 143.
Thubana microcera  Gozmány, 1978: 236, syn. n.

#### Material examined.

China, Zhejiang Province: 13 ♂, Wuyanling, Taishun (27°33'N; 119°42'E),790 m, 2–3.VIII.2007, coll. Qing Jin; 2 ♂, 2 ♀, same locality, 680 m, 930 m, 28–31.VII.2005, Yunli Xiao; 4 ♂, 2 ♀, Tianmushan (30°26'N; 119°34'E), Lin’an, 350 m, 7–8.VIII.2007, coll. Qin Jin, 10 ♂, 800 m, 19.VIII.1999, coll. Houhun Li et al., 5 ♂, 2 ♀, 500 m, 16.VIII.1999, coll. Houhun Li et al.; 1 ♂, Qingliangfeng (30°07'N; 118°51'E), Lin’an, 900 m, 12.VIII.2005, coll. Yunli Xiao. Anhui Province: 4 ♂, Mozitan, Huoshuan (31°24'N; 116°19'E), 12.VIII.2004, coll. Jiasheng Xu & Jialiang Zhang; 5 ♂, Huangshan (29°43'N; 118°18'E), 6–7.VIII.2004, coll. Jiasheng Xu and Jialiang Zhang; 6 ♂, Jiuhuashan (30°23'N; 117°48'E), 8–9.VIII.2004, coll. Jiasheng Xu & Jialiang Zhang. Fujian Province: 2 ♂, Wuyishan (26°54'N; 116°42'E), 740 m, 19–24.V.2004, coll. Haili Yu; 1 ♂, Qingyunshan, Yongtai (25°52'N; 118°57'E), 550 m, 18.IX.2002, coll. Xinpu Wang. Jiangxi Province: 4 ♂, Xiaoxidong, (26°57'N; 114°17'E), 1,3–4.VII.1978; 2 ♂, Tonggu (28°32'N; 114°22'E), 28.VII.1982, 10.V.1983; 3 ♀, Ciping (26°34'N; 114°10'E), 13.VII.1978; 1 ♂, 1 ♀, Xiashan, Yichun (27°47'N; 114°23'E), 7,30.VII.1980; Jinpenshan (29°20'N; 117°00'E), 2 ♂, 18–19.VIII.2006, coll. Jiasheng Xu & Weichun Li. Henan Province: 3 ♂, 1 ♀, Xiaguan, Neixiang (33°02'N; 111°50'E), 650 m, 10,12.VII.1998, coll. Houhun Li; 1 ♀, Huangshi’an (33°40'N; 111°37'E), Xixia, 890 m, 19.VII.1998, coll. Houhun Li. Hunan Province: 1 ♂, Zhangjiajie (29°49'N; 110°26'E), 650 m, 7.VIII.2001, coll. Houhun Li & Xinpu Wang. Hubei Province: 3 ♂, 2 ♀, Maoba (30°02'N; 109°02'E), Lichuan, 700 m, 28–29.VII.2007, coll. Houhun Li et al. Guizhou Province: 1 ♂, Jiangkou (27°41'N; 108°50'E), 600 m, 27.VII.2001, coll. Houhun Li & Xinpu Wang.

**Figures 11-14:**
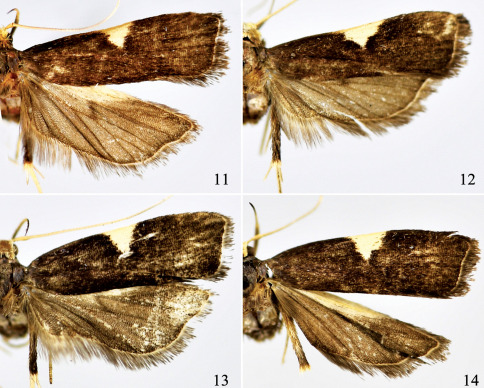
Adults of Thubana leucosphena Meyrick, showing variation of costal patch (11–12 >, 13–14 +).

**Figures 15-16:**
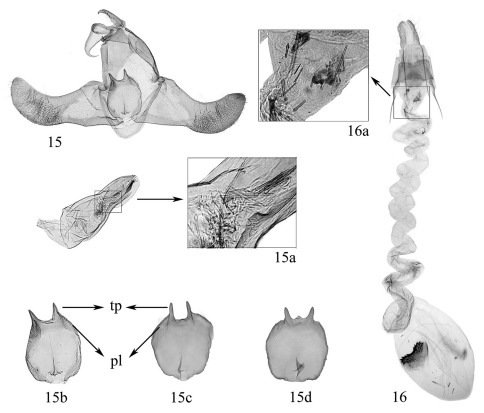
Thubana leucosphena Meyrick. **15** male genitalia **15a** aedeagus, showing spicules **15b–d** variation of juxta (**pl** = posterolateral lobes, **tp** = thornlike processes; slide Nos. **b**: ZYM06315, **c**: ZYM06321, **d**: ZYM06179) **16** female genitalia **16a** ductus bursae, showing spicules on inner surface, slide No. ZYM06193.

#### Diagnosis.

This species is characterized by the following characters: male genitalia (Fig. 15) with juxta having a lobulate projection at middle near anterior margin and two large thornlike processes on caudal margin, the posterolateral lobes inconspicuous (Fig. 15b-d), the aedeagus shorter than valva, the cornuti consisting of a long slender band, a bundle of brushlike spines, a dentate plate, and sometimes with a few dispersed deciduous spicules (Fig. 15a); female genitalia (Fig. 16) with apophysis anterioris about 1/2 length of apophysis posterioris, the caudal margin of 8th sternite slightly emarginate at middle, the ductus bursae long, twisted, with spicules on inner surface (Fig. 16a), and the signum spinulose, semiovate, slightly emarginate at upper margin. Thubana leucosphena is very close to Thubana felinaurita, but differs as noted in the description of the latter.

#### Distribution.

China (Anhui, Fujian, Jiangxi, Henan, Hunan, Hubei, Guizhou, Zhejiang).

#### Discussion.

This species was described by Meyrick (1931) based on three specimens collected from Guanxian of Sichuan Province in China: “two males” and “a third example”. Clarke (1965) rectified the “two males” as Oecophoridae and chose the “third example”, a female, as the lectotype of Thubana leucosphena. Meyrick (1935) mentioned the occurrence of this species in Tianmushan of Zhejiang Province. Gozmány (1978) described Thubana microcera on the basis of a male specimen collected from Tianmushan and noticed that itcould be distinguished from Thubana leucosphena by the shape of the costal patch. In this study, however, we noticed that the costal patch varies from triangular to trapezoidal both within male specimens of Thubana microcera (Figs 11–12) and female specimens of Thubana leucosphena (Figs 13–14). We also found that males collected from Zhejiang, Jiangxi, Hubei and Henan provinces match with those of Thubana microcera described by Gozmány, females match with those of Thubana leucosphena. Besides, we observed the deciduous spicules from the male aedeagus in the ductus bursae of Thubana leucosphena. What is more, no other species of Thubana were collected in these localities so far. Hence, we treat Thubana microcera as a junior synonymof  Thubana leucosphena, and regard the variation of the shape of costal patch from triangular to trapezoidal as intraspecific variation.

#### Notes.

The previous description did not mention the median projection on posterior margin of the juxta. Though this projection (Fig. 15b) is not present in most individuals, we found it present in some males, either inconspicuous (Fig. 15c) or small but visible (Fig. 15d). We consider this variation as intraspecific because other characters fit well with Thubana leucosphaena.

## Supplementary Material

XML Treatment for Thubana

XML Treatment for Thubana albinulla

XML Treatment for Thubana albiprata

XML Treatment for Thubana albisignis

XML Treatment for Thubana bathrocera

XML Treatment for Thubana deltaspis

XML Treatment for Thubana dialeukos

XML Treatment for Thubana leucosphena

XML Treatment for Thubana xanthoteles

XML Treatment for Thubana felinaurita

XML Treatment for Thubana felinaurita

XML Treatment for Thubana dialeukos

XML Treatment for Thubana xanthoteles

XML Treatment for Thubana leucosphena
